# SNP Discovery and Development of a High-Density Genotyping Array for Sunflower

**DOI:** 10.1371/journal.pone.0029814

**Published:** 2012-01-04

**Authors:** Eleni Bachlava, Christopher A. Taylor, Shunxue Tang, John E. Bowers, Jennifer R. Mandel, John M. Burke, Steven J. Knapp

**Affiliations:** 1 Center for Applied Genetic Technologies, University of Georgia, Athens, Georgia, United States of America; 2 Department of Plant Biology, University of Georgia, Athens, Georgia, United States of America; University of Umeå, Sweden

## Abstract

Recent advances in next-generation DNA sequencing technologies have made possible the development of high-throughput SNP genotyping platforms that allow for the simultaneous interrogation of thousands of single-nucleotide polymorphisms (SNPs). Such resources have the potential to facilitate the rapid development of high-density genetic maps, and to enable genome-wide association studies as well as molecular breeding approaches in a variety of taxa. Herein, we describe the development of a SNP genotyping resource for use in sunflower (*Helianthus annuus* L.). This work involved the development of a reference transcriptome assembly for sunflower, the discovery of thousands of high quality SNPs based on the generation and analysis of ca. 6 Gb of transcriptome re-sequencing data derived from multiple genotypes, the selection of 10,640 SNPs for inclusion in the genotyping array, and the use of the resulting array to screen a diverse panel of sunflower accessions as well as related wild species. The results of this work revealed a high frequency of polymorphic SNPs and relatively high level of cross-species transferability. Indeed, greater than 95% of successful SNP assays revealed polymorphism, and more than 90% of these assays could be successfully transferred to related wild species. Analysis of the polymorphism data revealed patterns of genetic differentiation that were largely congruent with the evolutionary history of sunflower, though the large number of markers allowed for finer resolution than has previously been possible.

## Introduction

Until recently, a major limitation in the genetic dissection of complex traits in both plants and animals has been the lack of availability of large numbers of genetic markers that can be assayed in an efficient manner. When combined with rapid advances in next-generation sequencing technologies, however, the high levels of single nucleotide polymorphism (SNP) diversity that are present in most plant and animal gene pools (e.g., [1]–[5]) have made possible the development of high-throughput genotyping platforms. These platforms, which allow for the simultaneous interrogation of thousands of SNPs from throughout the genome, have the potential to facilitate the rapid development of high-density genetic maps, and to enable genome-wide association studies as well as molecular breeding approaches in a variety of taxa.

The development of high-throughput SNP genotyping assays requires large-scale SNP discovery. Moreover, for such assays to be generally useful, the SNPs should be selected to represent the diversity present across the target gene pool, as opposed to being population specific. This, in turn, requires large amounts of sequence data from a diverse panel of individuals. While the necessary genomic resources already exist for a number of species (e.g., [6]–[8]), many others species lack the resources required for the development of such tools. Here we describe the results of a large-scale sequencing and SNP discovery effort aimed at developing a high-density SNP genotyping array in one such species – sunflower (*Helianthus annuus* L.).

Sunflower is a globally-important oilseed crop that is grown on ca. 25 million hectares per year. Due to the economic and ecological importance of cultivated and wild sunflower, *H. annuus* has emerged as a model species for genetic and genomic studies in the Compositae, which is one of the largest and most diverse families of flowering plants. While great strides have been made in the development of sunflower genomic resources [reviewed in 9], relatively few SNP markers have been developed, and even fewer have been placed on the sunflower genetic map [10]. Nevertheless, previous studies have revealed high levels of SNP diversity across the cultivated sunflower gene pool, and population genetic analyses have suggested that patterns of linkage disequilibrium in modern cultivars are appropriate for high-resolution association mapping [11]–[13].

In this study, we describe the development of a sunflower reference transcriptome assembly based primarily on long-read ESTs, as well as the generation and analysis of short-read transcriptome data from numerous accessions chosen to represent the diversity within the sunflower gene pool. This work resulted in the production of ca. 6 Gb of next-generation sequence data which was then compared against the reference transcriptome assembly for the purposes of SNP discovery. We identified thousands of high-quality SNPs using a customized bioinformatics pipeline, and selected the best set of 10,640 SNPs for the development of a high-throughput SNP genotyping array using Illumina's iSelect Infinium platform. We then used this SNP array to screen a diverse panel of sunflower accessions as well as a pair of related species. The results of this work revealed a high frequency of polymorphic SNPs and relatively high levels of transferability of assays across species, making this SNP array an ideal tool for the genetic analysis of sunflower and related species.

## Materials and Methods

### *De novo* reference transcriptome assembly

In order to build a reference transcriptome assembly, we started by combining publicly-available long-read ESTs from elite, inbred sunflower lines (RHA280, RHA801, HA89, HA300b, PSC8, and EMIL; Table 1) with next-generation sequences obtained from sunflower line HA89 using the 454 GS FLX platform (Roche, Indianapolis, IN) and used them to build a *de novo* transcriptome assembly using MIRA [14]. These lines represent the two primary heterotic groups within the sunflower gene pool (i.e., the restorer [RHA] and maintainer [HA] lines). The long-read ESTs, which included paired-end reads of HA89, were produced primarily by the Compositae Genome Project (CGP; http://cgpdb.ucdavis.edu/) via Sanger sequencing and subsequently deposited in GenBank (http://www.ncbi.nlm.nih.gov/dbEST/). In contrast, we produced the 454 data from HA89 by extracting RNA from developing seeds, roots, disc florets, and leaves using TRIZOL Reagent (Invitrogen, Carlsbad, CA) according to the manufacturer's guidelines, preparing cDNA using the SuperScript III Reverse Transcriptase kit (Invitrogen), pooling and normalizing the cDNA using the TRIMMER-DIRECT kit (Evrogen, Moscow, Russia), and sequencing the resulting normalized pool using a Roche 454 GS FLX instrument with XLR (Titanium) sequencing chemistry. This sequencing was performed at the Georgia Genomics Facility at UGA.

**Table 1:** Summary of DNA sequence data used in the reference assembly (above line) and SNP discovery (below line).

Sunflower Line	Accession ID	Sequencing Method	Read Length (range in bp)	Read Length (avg. in bp)	Number of Reads	Total Sequence (Mb)
RHA280	PI 552943	Sanger	100–809	397	20,892	8.3
RHA801*	PI 599768	Sanger	100–814	425	22,603	9.6
HA89	PI 599773	Sanger	100–923	712	39,569	28
HA300b	n/a	Sanger	85–546	354	1,485	0.5
PSC8	n/a	Sanger	100–922	478	15,837	7.6
EMIL	n/a	Sanger	101–625	356	2,169	0.8
ANN1238	n/a	Sanger	100–1013	713	27,957	30
HA89*	See above.	454 GS FLX XLR	50–622	285	66,851	19
RHA373	PI 560141	Illumina	36	36	21,601,273	777.7
RHA415	PI 607506	Illumina	36	36	11,341,180	408.3
HA383	PI 578872	Illumina	36	36	12,717,269	457.8
HA434	PI 633744	Illumina	36	36	25,661,886	923.8
RHA455	PI 642774	Illumina-PE	2×90	180	4,673,377	841.2
RHA468	n/a	Illumina-PE	2×90	180	4,459,305	802.7
HA89	See above.	Illumina-PE	2×90	180	4,943,677	889.9
HA412-HO	PI 642777	Illumina-PE	2×90	180	3,690,226	664.2
				Total	89,245,173	5,869.40

*These sequence datasets were used for both the reference assembly and for SNP identification.

To obtain a high quality transcriptome assembly using MIRA, we set a minimum overlap score equal to 20 and an 85% minimum match between two reads to be considered for assembly without increasing the penalty for alignments containing long gaps. We also allowed the skim algorithm to be called in-between each main pass, turned off the genomic pathfinder algorithm, as suggested for EST assemblies, and used a base quality of reads equal to 20. Further, we allowed identification of possible sequencing vector relics at the start of the sequence and clipping away a maximum of 25 bp. The aforementioned parameters were selected empirically after the evaluation of multiple assemblies in an attempt to optimize the total number of contigs as well as the number of contigs that included HA89 Sanger paired-end reads from the CGP data. To evaluate the quality of each unigene set, we counted the number of contigs containing both the 5′ and 3′ ends of the HA89 paired-end Sanger reads. When this number increased, we visually inspected hundreds of contig alignments to confirm that the increase was not due to misassembly of paralogs. When 5′ and 3′ paired-end reads of HA89 could not be assembled into a single contig, either due to poor sequence quality at the end of the reads or lack of complete coverage of long transcripts, they were outputted as singletons with identical names followed by a unique identifier. Because these singleton pairs come from opposite ends of the same gene, they were effectively treated as a single locus for the purposes of SNP selection (see below). Finally, long-read ESTs generated from wild sunflower (ANN1238) by the CGP were separately assembled into contigs and singletons with TGICL [15] using the default parameters of 40 bp minimum length of overlap and 95% minimum identity of overlap.

### Transcriptome re-sequencing and alignment of short reads

Transcriptome re-sequencing data was obtained from multiple elite, inbred sunflower oilseed lines representing the two major heterotic groups, including: RHA373, RHA415, RHA455, RHA468, HA89, HA383, HA412-HO, and HA434 (Table 1). For RHA373 and RHA415, total RNA was extracted from developing seeds, roots, disc florets, and leaves using TRIZOL Reagent (Invitrogen, Carlsbad, CA) according to the manufacturer's guidelines. These samples were then sequenced separately with one tissue/genotype being run in each of the eight flow cell channels on a single Illumina Genome Analyzer (Illumina Inc., San Diego, CA) run with 36 bp single-end reads. Similarly, RNA of HA383 and HA434 was extracted from developing seeds, roots, disc florets, and leaves as above and cDNA was prepared using the SuperScript III Reverse Transcriptase kit (Invitrogen). The cDNA derived from each of the four tissues was then pooled and normalized using the TRIMMER-DIRECT kit (Evrogen, Moscow, Russia); following normalization, the cDNA was size-selected (>650 bp) via agarose gel electrophoresis. Each of the normalized cDNA pools (one per genotype) was sequenced in four of the eight flow cell channels in a single Illumina GA run with 36 bp single-end reads. For RHA455, RHA468, HA89, and HA412-HO, RNA was extracted from developing seeds, roots, disk florets, and leaves as described above, bulked across tissues, and each genotype was then sequenced in two of the eight flow cell channels of an Illumina GA run with 2×90 bp paired-end reads. All Illumina sequencing was performed at the National Center for Genome Resources in Santa Fe, NM.

These short-read ESTs, along with the long-read ESTs derived from RHA801 and HA89, were then mapped onto the reference sequence scaffold using MOSAIK (Michael Strömberg, Boston College) for SNP discovery. Pairwise alignment of reads with the reference sequence was performed with the MosaikAligner module using the alignment algorithm “all” to store all hash positions per seed. The alignment mode “unique,” which places only uniquely aligned reads onto the reference, was used for the Illumina, Sanger, and GS FLX reads, while the alignment mode “all,” which finds all possible alignments (as suggested for resolving paired-end reads) was used for the Illumina paired-end data. We used a hash size of 14 for Sanger and GS FLX Titanium reads and hash size of 15 for Illumina reads. We allowed 4 and 12 mismatches for Illumina and Illumina paired-end reads, respectively. For the Sanger and GS FLX reads, we allowed up to 5% mismatches and a maximum of 100 hash positions per seed. Multiple sequence alignment was conducted with the MosaikSort module using only uniquely aligned Illumina, Sanger, and GS FLX reads, while for Illumina paired-end reads we allowed sorting of orphaned unique reads when one of the two paired-end mates could not be uniquely aligned, and we ignored all reads when both paired-end mates were not-uniquely aligned. The MosaikAssembler module was used to produce a final multiple sequence alignment in an assembly format.

### SNP discovery using a customized bioinformatics pipeline

We mined SNPs from the Illumina-Sanger-GS FLX contigs with a minimum of 6 reads and a maximum of 5000 reads using the following criteria: (i) a minimum of two reads (either short- or long-read ESTs) per genotype at the SNP position, (ii) a minimum of two distinct genotypes at the SNP position that satisfy criterion (i), and (iii) a minimum allele frequency of 0.9 at the SNP position of interest within each given genotype. We also used a tblastx-based intron finding Perl script (http://int-citrusgenomics.org/usa/ucr/Files.php) with updated *Arabidopsis* genome sequence information available from TAIR (http://www.arabidopsis.org/) to discard SNPs in regions spanning putative introns in sunflower unigenes.

We deposited the results of our SNP discovery analysis in a modified database built on the MAGIC interface [16] that was redesigned to accommodate next-generation sequencing data (http://sourceforge.net/projects/ngmagic/). This database allows for filtering of reference unigenes according to their name, length, and depth (number of assembled sequences), displays the aligned contig corresponding to each unigene, and highlights sequence variants (i.e., SNPs and INDELs) for each genotype. Sequence variants can also be filtered using multiple criteria, such as unigene name, genotype identity, class (SNP or INDEL), number of reads at the variant position, and major allele frequency. Moreover, a blast tool permits blastn and tblastn to the reference unigenes for the identification of unigenes with sequence similarity to candidate genes and the development of SNP assays that will allow genetic mapping of these genes. During the SNP discovery analysis, custom scripts were also used to improve the quality of the reference assembly using short-read sequence information from contigs with sufficient depth to correct ambiguous nucleotides in the reference scaffold.

### Functional annotation of sunflower unigenes

Nucleotide sequences from all unigenes used for SNP discovery were translated in all six reading frames and the length of the longest open reading frame was recorded. These sequences were also blasted against all predicted proteins from nine fully-sequenced genomes, including: *Arabidopsis thaliana* (L.) Heynh, *Ricinus communis* L. (castor), *Lotus japonicus* L., *Medicago truncatula* Gaertn., *Carica papaya* L. (papaya), *Populus trichocarpa* Torr. & Gray (poplar), *Sorghum bicolor* (L.) Moench, *Solanum lycopersicum* L. (tomato) and *Vitis vinifera* L. (grape vine). All unigenes with hits (at an E-value threshold 1e-06) to proteins from at least one of the nine plant genomes were considered putative “genes” if their predicted open reading frame (ORF) was longer than 50 amino acids, and “pseudogenes or gene fragments” if their predicted ORF was shorter than that value. Unigenes that had no similarity with putative proteins from any of the nine genomes were classified as “possible novel genes” if their predicted ORF was longer than 75 amino acids, and “not genes” (presumably UTR sequences) if their predicted ORF was shorter than that value. These length thresholds (i.e., 50 and 75 amino acids) were empirically determined based on the size distributions of the longest predicted ORFs that either matched or did not match putative genes from the nine sequenced species.

Sequences of the 10,640 unigenes selected for inclusion on the Infinium Beadchip (see below for details) were next imported into Blast2GO (http://www.blast2go.de/) [17], [18] for automated functional annotation. We blasted the unigene dataset against the NCBI nr database with default parameters (E-value threshold 1e-03) using the blastx program in the QBlast mode. Mapping of homologue sequences to GO terms and GO term assignment was performed using the default parameters, which were an E-value hit filter of 1e-06, annotation cut-off of 55, and GO weight of 5. Sequences that could not be annotated using the above settings were re-annotated using an annotation cut-off of 45. These annotations were further augmented using the Annex-function of the GO Annotation Toolbox (http://www.goat.no/) [19]. InterProScan terms were obtained for all unigenes [20], and Kegg pathway maps (http://www.genome.jp/kegg/pathway.html) [21] were downloaded for all enzyme codes.

### Selection of SNPs for array development

From the initial 85,063 SNP dataset (see Results and Discussion), we selected 10,640 SNPs with Illumina Infinium probe design scores ≥0.70 and GoldenGate probe design scores ≥0.55 to interrogate SNPs that can be also converted into GoldenGate assays. Because [A/T] and [C/G] SNPs require two Illumina bead types (probes), and because we were trying to maximize the number of unique SNPs on the array, we discarded [A/T] and [C/G] SNPs and only designed probes for [A/C] ( = [T/G]) and A/G ( = [T/C]) SNPs. We also discarded SNPs in unigenes that were annotated as “not genes” and “pseudogenes or gene fragments” to exclude SNPs in unigenes with short ORFs that may not represent functional genes. We then tabulated the number of nucleotide differences in the regions flanking each SNP (i.e., across the entire contig), excluding the SNP itself, for short and long reads aligned to reference unigenes to infer misaligned contigs or excess sequencing errors. This metric, which was initially derived for each genotype separately, was summed across genotypes and was divided by the number of reads aligned at each SNP position. SNPs with values larger than 10 were discarded, while more than 80% of the SNPs had values of 3 or lower. Next, we selected a single SNP per unigene taking into consideration the Infinium design scores and biasing the selection towards those SNPs that were supported by sequence data from multiple genotypes. Over 80% of the selected SNPs were supported by sequence data from at least four genotypes. For the HA89 Sanger paired-end reads that could not be assembled into a contig, a single SNP was retained from just one of the two members of each singleton pair. After reducing the dataset to include just a single SNP per unigene, we retained the 10,640 SNPs with the highest Infinium design scores.

### SNP genotyping

Infinium Beadchips were manufactured by Illumina in a 24×1 format and used to genotype a panel of 36 accessions including oilseed and confectionery (i.e., non-oil) cultivars, landraces, wild *H. annuus*, and 2 individuals each of *H. argophyllus* and *H. niveus* ssp. *tephrodes* (Table 2). The oilseed and confectionery cultivars included inbred RHA and HA lines as well open-pollinated (i.e., non-inbred) cultivars. *Helianthus argophyllus* and *H. niveus* ssp. *tephrodes* are relatively close congeners of sunflower that are of interest to breeders as possible sources of exotic alleles. Samples of three of these 36 accessions (RHA415, HA370, RHA468) were included twice as controls to assess the repeatability of allele calls. For each accession, DNA was extracted from fresh or lyophilized leaf tissue using a modified CTAB method [22] and DNA concentrations were quantified using the Quant-iT PicoGreen dsDNA reagent (Invitrogen). Genotyping was conducted according to the manufacturer's recommendations for Infinum II assay workflow. Beadchips were analyzed on Illumina's iScan System at the Emory University Biomarker Service Center. Prior to hybridization of the Beadchips, DNA was diluted to 50 ng/ul and quality was assessed using a BioTek Synergy HT Microplate Spectrophotometer (BioTek Instruments, Winooski, VT) and agarose gel electrophoresis. All SNP data analyses were conducted using GenomeStudio ver. 2009.1 (Illumina). Briefly, intensity data were loaded in GenomeStudio and clusters were generated using a GenCall score cutoff of 0.15, as recommended by Illumina for Infinium products. After auto-clustering of the data, SNP clusters were manually reviewed and edited as appropriate to refine cluster positions, and SNP calls were exported for analysis.

**Table 2:** Summary of sunflower lines/accessions genotyped using the SNP array.

Sunflower Line	Species	Accession ID	Type
ANN1238	*H. annuus*	n/a	Wild (Nebraska)
ANN1811	*H. annuus*	PI 494567	Wild (Texas)
Arikara	*H. annuus*	PI 369357	Native American Landrace
Havasupai	*H. annuus*	PI 369358	Native American Landrace
Hopi	*H. annuus*	PI 369359	Native American Landrace
Seneca	*H. annuus*	PI 369360	Native American Landrace
Mennonite	*H. annuus*	PI 650650	Open-Pollinated; Non-Oil
Shemesh	*H. annuus*	n/a	Open-Pollinated; Non-Oil
Peredovik	*H. annuus*	PI 650338	Open-Pollinated; Oil
Pervenets	*H. annuus*	PI 483077	Open-Pollinated; Oil
VNIIMK8931	*H. annuus*	PI 340790	Open-Pollinated; Oil
RHA280	*H. annuus*	PI 552943	RHA Non-Oil
HA292	*H. annuus*	PI 552937	HA Non-Oil
RHA274	*H. annuus*	PI 599759	RHA Oil
RHA373	*H. annuus*	PI 560141	RHA Oil
RHA409	*H. annuus*	PI 603990	RHA Oil
RHA415*	*H. annuus*	PI 607506	RHA Oil
RHA417	*H. annuus*	PI 600000	RHA Oil
RHA455	*H. annuus*	PI 642774	RHA Oil
RHA468*	*H. annuus*	n/a	RHA Oil
RHA801	*H. annuus*	PI 599768	RHA Oil
NMS373	*H. annuus*	PI 560141	RHA Oil
NMS377	*H. annuus*	PI 560145	RHA Oil
HA89	*H. annuus*	PI 599773	HA Oil
HA342	*H. annuus*	PI 509052	HA Oil
HA370*	*H. annuus*	PI 534656	HA Oil
HA372	*H. annuus*	PI 534658	HA Oil
HA383	*H. annuus*	PI 578872	HA Oil
HA407	*H. annuus*	PI 597371	HA Oil
HA412-HO	*H. annuus*	PI 642777	HA Oil
HA434	*H. annuus*	PI 633744	HA Oil
HA821	*H. annuus*	PI 599984	HA Oil
ARG1820	*H. argophyllus*	PI 494580	Wild Relative
ARG1834	*H. argophyllus*	PI 494582	Wild Relative
NIV20	*H. niveus* ssp. *tephrodes*	PI 650020	Wild Relative
NIV58	*H. niveus* ssp. *tephrodes*	PI 613758	Wild Relative

*These DNA samples were genotyped twice each to assess repeatability of genotype calls.

### Diversity analyses

In order to assess the utility of the SNP array for genetic analyses in diverse germplasm, the resulting data were analyzed in a population genetic framework and used to investigate genetic differentiation amongst the genotyped accessions. Gene diversity (i.e., expected heterozygosity) for each SNP was estimated using GenAlEx v. 6.1 [23]. Genetic differentiation amongst the surveyed accessions was then investigated using the Bayesian, model-based clustering algorithm implemented in the software package STRUCTURE [24]. Briefly, individuals were assigned to *K* population genetic clusters based on their multi-locus genotypes. Clusters were assembled to minimize intra-cluster Hardy-Weinberg and linkage disequilibrium and, for each individual, the proportion of membership in each cluster was estimated. This analysis did not rely on prior population information (i.e., USEPOPINFO was turned off). For each analysis, *K* = 1-12 population genetic clusters were evaluated with 5 runs per *K* value. After checking to ensure that the results of each run were in general agreement, the probability values were averaged across runs for each cluster. For each run, the initial burn-in period was set to 50,000 with 100,000 MCMC iterations. The most likely number of clusters was determined using the Delta*K* method of Evanno *et al.* [25]. Genetic relationships amongst accessions were also explored graphically via principal coordinates (PCO) analysis using GenAlEx. For this analysis, a standard genetic distance [26] matrix was first constructed based on the multi-locus genotypes. This matrix was then used for the PCO analysis, and the first two principal coordinates were graphed in two-dimensional space.

## Results and Discussion

### Reference assembly and SNP discovery

As noted above, the reference transcriptome assembly was based on a large collection of Sanger and 454 sequences from cultivated sunflower as well as Sanger sequences from wild sunflower (ANN1238) (Table 1). After elimination of unigenes with at least 95% similarity over 90% of their sequence length, the reference assembly coalesced into 50,020 unigenes, with 19,486 contigs (average length 828.5 bp) and 30,534 singletons (average length 444.2 bp). The reference assembly is available for download as Dataset S1.

After discarding sequences that did not meet the criteria outlined in the Materials and Methods, a total of 85,063 SNPs identified from 18,053 unigenes were retained for further analysis (see Supporting Information S1 for additional details). On average, these polymorphisms occurred at a rate of 1 SNP per 163.6 bp, roughly on par with the frequency of SNPs previously seen in sunflower [12], [13], and there was an average of 62.8 reads covering each SNP position. As has been observed in other plant and animal genomes (e.g., [27], [28]) [A/T] and [C/G] SNPs were less common than other types in the sunflower transcriptome (i.e., transitions were more common that transversions; Figure S1); as noted above, these were not converted into SNP assays. From this set of 85,063 SNPs, a total of 35,435 (41.7%) had quality scores above the required threshold for Infinium and GoldenGate probe design, and were used in the selection of the 10,640 SNPs that made it into the final Infinium Beadchip design. The design information for the full set of targeted SNP assays is available for download as Dataset S2.

### Functional annotation and SNP selection

After blasting the 37,545 sunflower unigenes used for SNP discovery against all predicted proteins of the nine sequenced plant genomes, 10,314 unigenes (24.5%) were found to have no significant hits, and were thus classified as “not genes” or “possible novel genes,” depending on the length of their predicted ORFs. The remaining 27,231 unigenes (72.5%) shared identity with proteins from at least one of the nine genomes, and 86.0% of these had hits with proteins in seven or more of the nine genomes. Of these, 1,237 were classified as putative “pseudogenes or gene fragments” due to their short predicted ORFs, resulting in 25,994 unigenes being classified as putative genes.

The subset of 10,640 unigenes that was ultimately selected for inclusion on the Infinium Beadchip, each of which came from a different unigene, included 8,229 unigenes with blast-based annotations augmented by InterProScan and Annex, 867 unigenes that had no blast hits to the NCBI nr database, 468 unigenes that had several blast hits, but which lacked GO-terms, and 1,076 unigenes that had blast hits and GO-terms, but no blast-based annotation for the selected parameters (e.g., they were hits to putative proteins). An average of 3.2 GO-terms (ranging from 1 to 27) were available for 9,305 of these unigenes. The 20 most abundant molecular functions, biological processes, and cellular components of the Gene Ontology vocabulary for the 10,640 unigenes are presented in Figure S2. We ultimately arrived at 26,767 blast-based annotations for 8,229 unigenes and 3,122 enzyme codes for 2,541 unigenes, and found InterProScan terms for 90 unigenes. Kegg maps were downloaded for 1,457 enzyme codes corresponding to 127 metabolic pathways. Annotation information for the final set of 10,640 genes targeted in the Infinium Beadchip design is provided in Dataset S3.

### SNP genotyping and genetic diversity analyses

Of the 10,640 targeted SNPs, 9,480 SNPs were included on the Infinium Beadchips due to a manufacturing loss of 10.9% of attempted SNPs, which was within the expected limits. For *H. annuus*, there were 7,970 SNPs that gave usable data for at least 80% of the individuals tested. Of these, 7,723 gave successful allele call data in both *H. annuus* and *H. argophyllus*, and 7,490 in both *H. annuus* and *H. niveus* ssp. *tephrodes*. Taken together, 7,381 of the SNPs gave usable data in all three species. Note that differences in the number of loci that worked in the *H. argophyllus* and *H. niveus* ssp. *tephrodes* were consistent with known phylogenetic relationships. That is, *H. argophyllus* is the sister species to *H. annuus* while *H. niveus* ssp. *tephrodes* resides in a different (sister) clade [29] and the former had a larger number of usable SNP assays compared to the latter. Importantly, for the three *H. annuus* DNA samples that were included twice as controls, the genotype calls differed for less than 2% of the SNPs. In all cases, these differences could be attributed to missing data in one or the other replicate, such that there were no instances in which different alleles were called between replicates.

In terms of polymorphism, 7,640 of the 7,970 SNPs that produced usable data in *H. annuus* were polymorphic. As expected, the modern breeding lines (i.e., RHA and HA lines) had significantly fewer loci scored as heterozygous when compared to the OPVs and landraces (13.4%±1.1% vs. 40.5%±4.0% [mean ± SE], respectively; *P*<0.01). Of the polymorphic loci, 6,692 of these had a minor allele frequency ≥10%. All subsequent genetic diversity analyses were performed on this reduced set of 6,692 SNPs. Gene diversity, or expected heterozygosity, calculated across the 32 *H. annuus* genotypes was 0.426±0.001 (mean ± standard error). After excluding the two wild *H. annuus* individuals, gene diversity dropped slightly to 0.424±0.001. This overall level of SNP diversity at polymorphic sites is comparable to previous estimates derived from the re-sequencing of PCR amplicons from cultivated sunflower accessions [12]. Note that since only two individuals were sampled within *H. argophyllus* and *H. niveus* ssp. *tephrodes*, these species were excluded from genetic diversity analyses.

**Figure 1:**
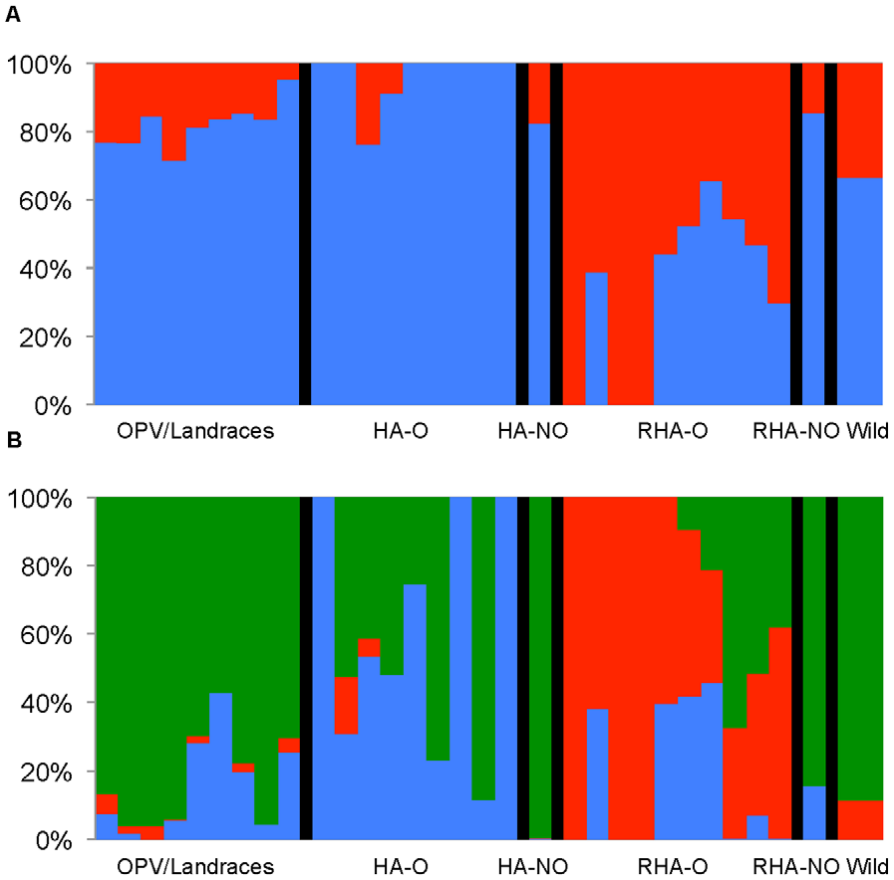
STRUCTURE results plot. Results of STRUCTURE analysis of the 32 *H. annuus* individuals based on all SNPs with MAF ≥0.10. A) Depicts the results for *K* = 2. B) Depicts the results for *K* = 3. Black bars represent dividers between the six groups: OPV/Landraces, HA-oil, HA-nonoil (HA-NO), RHA-oil, RHA-nonoil (RHA-NO), and wild *H. annuus*.

**Figure 2:**
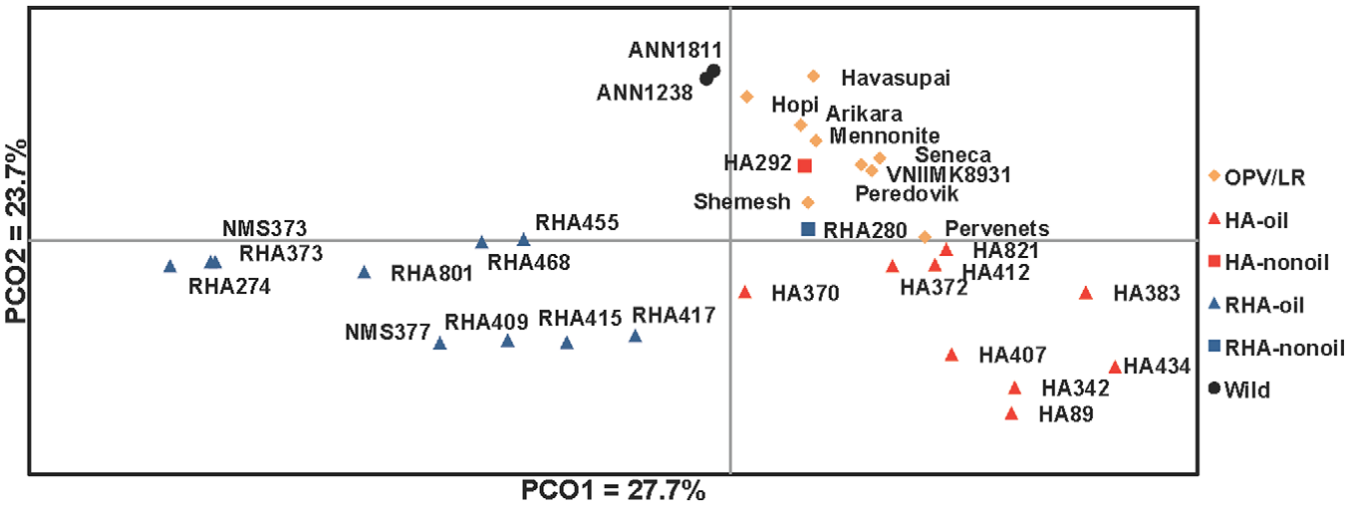
Principal coordinates analysis plot. Plot of the first two principal coordinates for the 32 *H. annuus* individuals based on all SNPs with MAF ≥0.10. Each data point represents an accession with one of six groups: OPV/Landraces (OPV/LR), HA-oil, HA-nonoil (HA-NO), RHA-oil, RHA-nonoil (RHA-NO), and wild *H. annuus*.

With regard to genetic differentiation in *H. annuus*, the Delta*K* method of Evanno *et al.* [25], indicated the presence of two genetically distinct clusters (i.e., *K* = 2; Figure 1; values of log likelihood and Delta*K* are reported in Figure S3), which largely corresponded to differentiation between the RHA oil lines and the balance of the lines surveyed. Increasing to *K* = 3 revealed additional differentiation, with the wild, OPV/landrace, and non-oil lines showing high membership in the same group, and the RHA and HA oil lines emerging as relatively distinct groups. Inspection of the PCO plot (Figure 2) reveals a relatively clear distinction between the RHA-oil lines and the balance of the *H. annuus* accessions along PCO1, which explains 27.2% of the total variation. PCO2, which explains 23.9% of the total variation, differentiates the wild and more primitive (i.e., landrace and OPV) accessions from the improved (i.e., RHA and HA) accessions. These results are generally consistent with previous population genetic analyses of the cultivated sunflower gene pool, where oilseed RHA lines have appeared to be relatively distinct from the balance of the gene pool [30]. However, the large number of loci surveyed in the present study appears to have allowed for finer resolution of the differences between the wild/primitive accessions and the improved accessions.

These overall patterns of genetic differentiation are largely congruent with the evolutionary history of sunflower as a crop plant. Following its domestication from wild sunflower in what is now the central United States, cultivated sunflower was first used as a source of edible seeds and for a variety of non-food applications (e.g., as a source of dye for textiles) [31], [32]. In the early 16^th^ century, however, it was taken to Europe by Spanish explorers where it was initially grown as an ornamental, but later became an important source of vegetable oil; breeding efforts thus focused increasingly on improving oil yield. Eventually, the germplasm that gave rise to the modern oilseed sunflower gene pool was brought back to North America and commercial production in the United States commenced in the 1960s, primarily using open-pollinated oilseed cultivars [33]. Shortly thereafter, however, attention turned to hybrid production, resulting in a focus on developing inbred lines within two primary heterotic groups that have been largely maintained as distinct breeding pools (i.e., the so-called R and B lines, represented by RHA and HA designations, respectively), and the resulting differentiation is readily apparent in the STRUCTURE plots and along PCO1.

### Future directions

Given our results, the high-density SNP array described herein appears to be an excellent resource for agricultural applications as well as evolutionary genetic studies in cultivated sunflower and its wild relatives. Indeed, the high level of polymorphism revealed by this array makes it an ideal tool for the development of a high-density genetic map of the sunflower genome, and the relatively high level of cross-species transferability of these assays suggest that it will also be a powerful tool for comparative genetic mapping studies aimed at understanding patterns of genome rearrangement between sunflower and related species. In addition, this array should prove useful for association mapping approaches aimed at correlating molecular polymorphisms with variation in phenotypic traits, as well as for molecular breeding approaches in sunflower. Finally, because our design criteria included GoldenGate probe design scores, the full set of SNPs and associated polymorphism data will provide researchers with a rich source of information for developing smaller, more targeted SNP arrays for a variety of applications.

## Supporting Information

Figure S1 **Frequency of SNP types based on the full set of 85,063 sunflower SNPs.**
(TIFF)

Figure S2 **The 20 most common GO terms in each of three categories for the 10,640 SNP-containing unigenes.** A) Biological Process. B) Molecular Function. C) Cellular Component.
(TIF)

Figure S3 **Log-likelihood and Delta** ***K*** **plots for the STRUCTURE analyses.** A) Log-likelihood plot. B) *DeltaK* plot.
(TIF)

Supporting Information S1 **Supporting Information for “SNP Discovery and Development of a High-Density SNP Genotyping Array for Sunflower.”**
(PDF)

Dataset S1 (RAR)

Dataset S2 (XLS)

Dataset S3 (XLS)
