# Supplementary figures and images for: SNP Discovery and Development of a High-Density Genotyping Array for Sunflower
Source: PLoS One. 2012 Jan 4;7(1):e29814. doi: 10.1371/journal.pone.0029814 (PMC3251610; doi:10.1371/journal.pone.0029814)

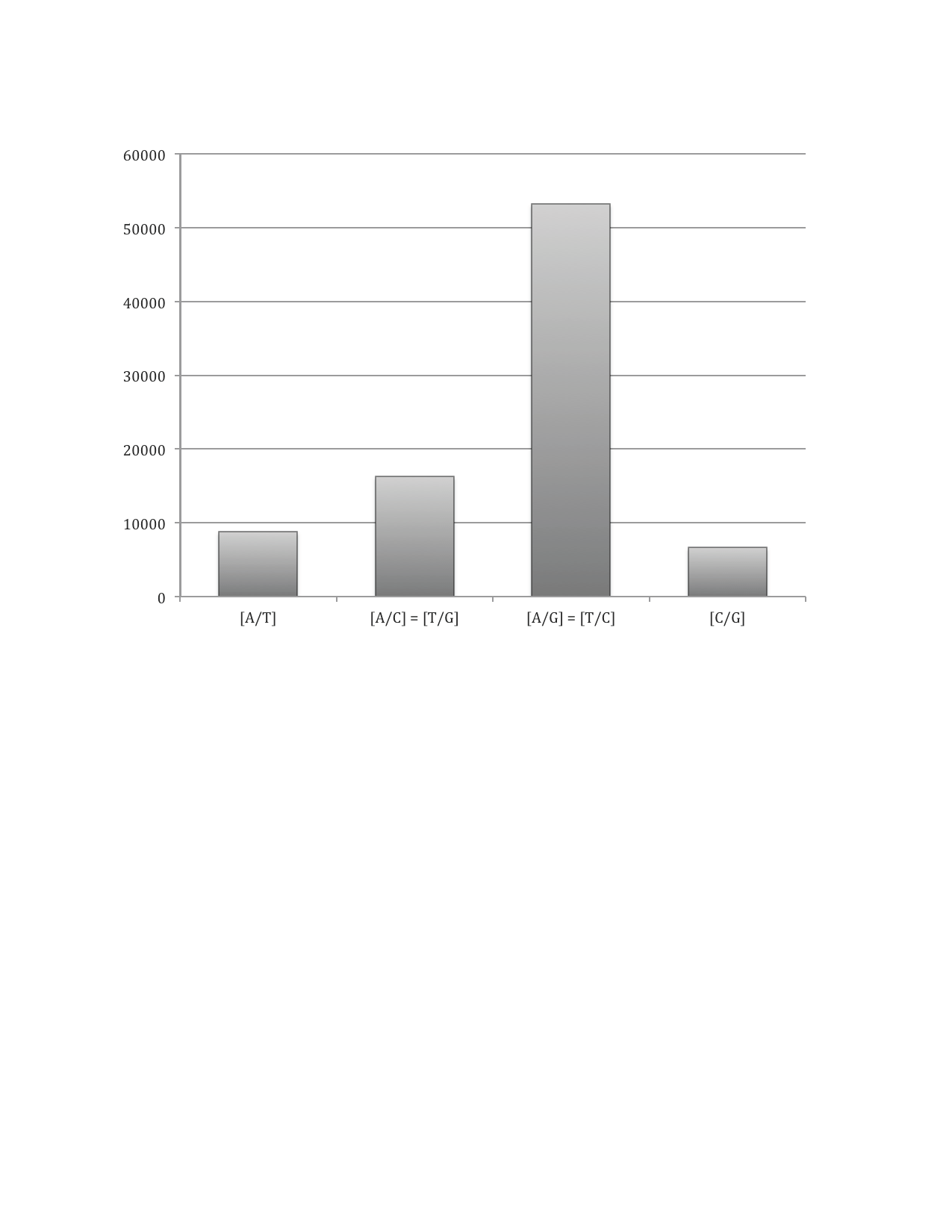

Supplement: Figure S1 — Frequency of SNP types based on the full set of 85,063 sunflower SNPs. (TIFF) [file pone.0029814.s001.tiff]

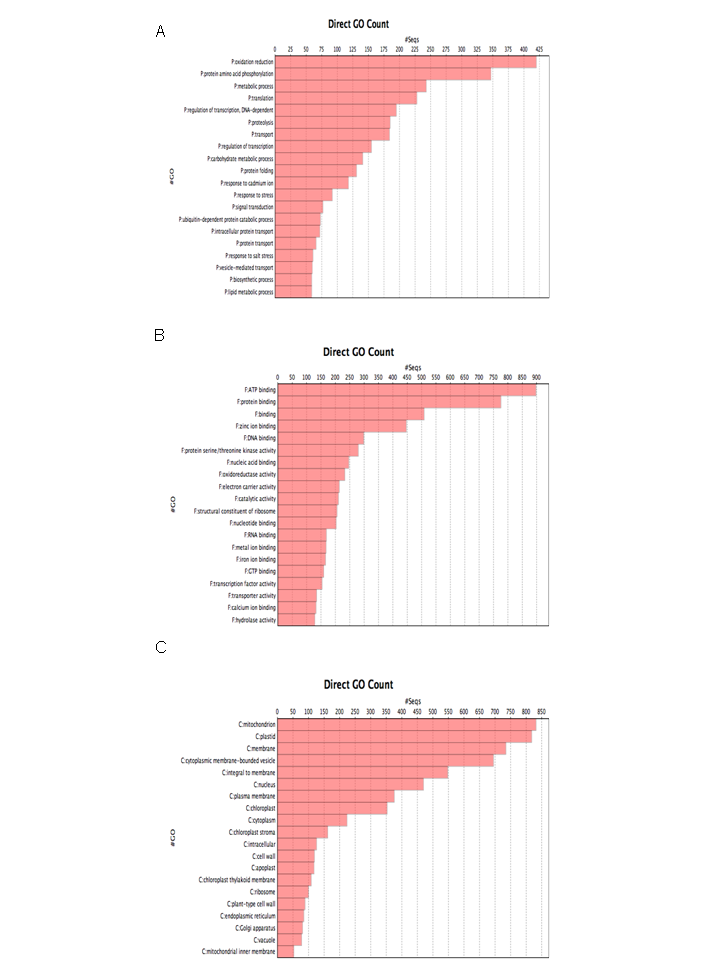

Supplement: Figure S2 — The 20 most common GO terms in each of three categories for the 10,640 SNP-containing unigenes. A) Biological Process. B) Molecular Function. C) Cellular Component. (TIF) [file pone.0029814.s002.tif]

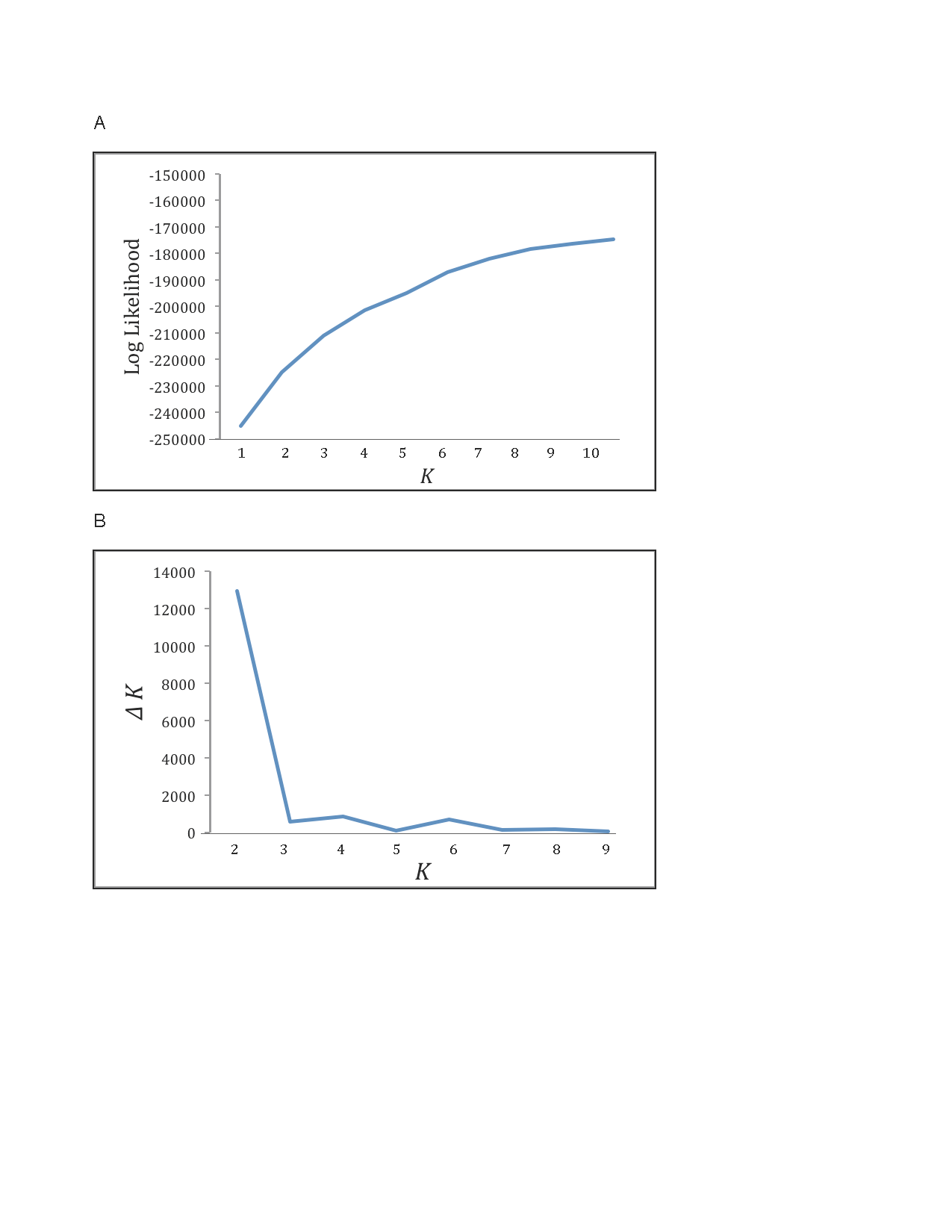

Supplement: Figure S3 — Log-likelihood and Delta K plots for the STRUCTURE analyses. A) Log-likelihood plot. B) DeltaK plot. (TIF) [file pone.0029814.s003.tif]
