# Supplementary material for: SNP Discovery and Development of a High-Density Genotyping Array for Sunflower
Source: PLoS One. 2012 Jan 4;7(1):e29814. doi: 10.1371/journal.pone.0029814 (PMC3251610; doi:10.1371/journal.pone.0029814)
Supplement: Supporting Information S1 — Supporting Information for “SNP Discovery and Development of a High-Density SNP Genotyping Array for Sunflower.” (PDF) [file pone.0029814.s004.pdf]

Supporting Information for "SNP Discovery and Development of a High-Density SNP Genotyping Array for Sunflower."

A total of 33,456,490 short reads were unambiguously mapped to 47,587 of these unigenes and a total of 37,545 unigenes with a minimum of 6 and a maximum of 5,000 reads were then used for SNP discovery. The limitation at the high end was employed because SNP identification with larger numbers of reads was too computationally intensive. These 37,545 unigenes comprised 17,316 contigs and 20,229 singletons from the reference assembly

A total of 85,747 SNPs satisfying the criteria outlined in the Materials and Methods were mined from the 37,545 unigenes; however, 684 SNPs that were flanking putative intron splice sites were discarded. Thus, a total of 85,063 SNPs identified in 18,053 unigenes were retained for further analysis. Notably, [A/G] SNPs (i.e., transistions) comprised 62.6% of the polymorphisms detected in sunflower transcriptome (Figure S1).

All 85,063 SNPs and their flanking sequences were evaluated using the Illumina Array Design Tool, resulting in the identification of 46,545 SNPs (54.7%) in 16,751 unigenes (92.8%) that had flanking regions amenable to successful Infinium probe design (i.e., Infinium quality score ≥ 0.55) and 47,643 SNPs (56.0%) in 16,096 unigenes (89.2%) that had flanking regions amenable to GoldenGate probe design (i.e., GoldenGate quality score ≥ 0.55). Ultimately, 35,435 SNPs (41.7%) in 15,087 unigenes (83.6%) had quality scores above the required threshold for both assays, and were used in the selection of the 10,640 SNPs that made it into the final Infinium Beadchip design. As noted in the Materials and Methods, the total pool of SNPs was ultimately narrowed to include on those with Infinium quality score ≥ 0.70 and GoldenGate quality score ≥ 0.55.
